# Synthetic lethality and synergetic effect: the effective strategies for therapy of IDH-mutated cancers

**DOI:** 10.1186/s13046-021-02054-x

**Published:** 2021-08-23

**Authors:** Kun Yao, Hua Liu, Jiajun Yin, Jianmin Yuan, Hong Tao

**Affiliations:** 1 Brain Science Basic Laboratory, The Affiliated Wuxi Mental Health Center with Nanjing Medical University, Wuxi, 214151 Jiangsu China; 2 Department of Clinical Psychology, The Affiliated Wuxi Mental Health Center with Nanjing Medical University, Wuxi, 214151 Jiangsu China; 3 Department of Pharmacy, The Second Affiliated Hospital of Soochow University, Suzhou, 215004 Jiangsu China

**Keywords:** Mutant IDH, Inhibitor, Drug resistance, Synthetic lethality, Synergetic therapy

## Abstract

Mutant isocitrate dehydrogenase 1/2 (mIDH1/2) gain a novel function for the conversion of *α*-ketoglutarate (*α*-KG) to oncometabolite R-2-hydroxyglutarate (R-2-HG). Two molecular entities namely enasidenib (AG-221) and ivosidenib (AG-120) targeting mIDH2 and mIDH1 respectively, have already been approved by FDA for the treatment of relapsed/refractory acute myeloid leukemia (R/R AML). However, the low responses, drug-related adverse effects, and most significantly, the clinically-acquired resistance of AG-221 and AG-120 has shown great influence on their clinical application. Therefore, searching for novel therapeutic strategies to enhance tumor sensitivity, reduce drug-related side effects, and overcome drug resistance have opened a new research field for defeating IDH-mutated cancers. As the effective methods, synthetic lethal interactions and synergetic therapies are extensively investigated in recent years for the cure of different cancers. In this review, the molecules displaying synergetic effects with mIDH1/2 inhibitors, as well as the targets showing relevant synthetic lethal interactions with mIDH1/2 are described emphatically. On these foundations, we discuss the opportunities and challenges for translating these strategies into clinic to combat the defects of existing IDH inhibitors.

## Background

**Fig. 1:**
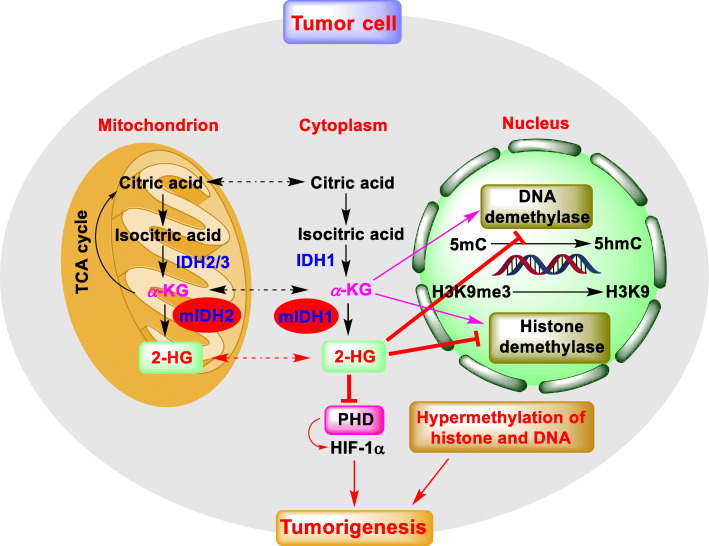
The presentation model for R-2-HG in tumorigenesis. R-2-HG produced by mIDH1/2 inhibits the α-KG dependent dioxygenases include DNA demethylase, histone demethylase, and PHD, leading to the hypermethylation of DNA and histone, and the increase of HIF-1α, respectively. These epigenetic alterations result in the blockade of cell differentiation and promote the initial engraftment and proliferation of tumor cells

IDH1/2 are metabolic enzymes that are responsible for catalyzing the conversion of isocitrate to *α*-KG in tricarboxylic acid cycle (TCA) [1]. Somatic mutations, such as R132H in IDH1 and R140Q or R172H in IDH2 have been found in various types of human cancers, including AML, glioma, chondrosarcoma and so on [2, 3]. Moreover, R132H accounts for more than 93% of IDH1 variants while R140Q and R172K mutants are predominant among all IDH2 variants in AML [4]. All these IDH mutants gain a neomorphic function for converting *α*-KG to R-2-HG. The accumulating R-2-HG acts as an *α*-KG analog to competitively inhibit *α*-KG dependent dioxygenases such as histone and DNA demethylases, resulting in DNA and histone hypermethylation (Fig. 1), which are responsible for the blockade of cell differentiation, and hence cancer development [5]. Also, R-2-HG can inhibit the activity of prolyl hydroxylase (PHD), which is an *α*-KG dependent dioxygenase focusing on the degradation of hypoxia-inducible factor 1α (HIF-1α), finally facilitate oncogenesis and tumor progression [5]. Interestingly, the stability of HIF-1α can also be regulated by *α*-KG. It was reported that the increased expression of HIF-1α is often detected in tumor cells with low levels of *α*-KG [6, 7].

**Fig. 2:**
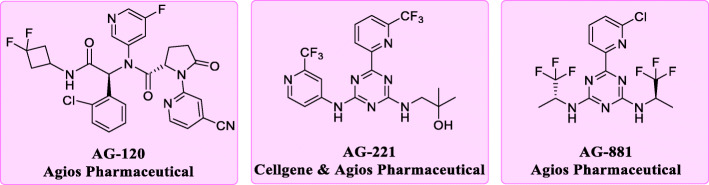
The representative structures of mIDH1/2 inhibitors. The phenyl glycine based AG-120 is an orally available mIDH1 inhibitor (IC_50_ [IDH1^R132H^] = 12 nM). The triazines based AG-221 is an orally available mIDH2 inhibitor (IC_50_ [IDH2^R140Q^] = 100 nM versus IC_50_ [IDH2^wild-type^] = 1.8 μM). AG-881 is a pan-IDH inhibitor against both mIDH1 and mIDH2 (IC_50_ [IDH1^R132H^] = 6 nM versus IC_50_ [IDH2^R140Q^] = 12 nM)

Currently, several inhibitors targeting IDH1/2 mutants have been reported [8, 9]. Among them, AG-221 and AG-120 are both first-in-class inhibitors approved by FDA in 2017 and 2018 respectively [10, 11]. Also, they are prescribed to treat adults suffering from R/R AML with IDH1/2 mutations. Vorasidenib (AG-881), a potent, oral, brain-penetrant dual inhibitor of both IDH1 and IDH2 mutants, is now undergoing several clinical tests (Fig. 2) [12].

Despite encouraging results were obtained from enasidenib and ivosidenib, their clinical applications remain limited because of several key factors. Firstly, the majority of AML patients treated with enasidenib have low responses (38.5% overall response rate, 20.2% complete remission) [13]. Worse results are identified in IDH-mutated glioma and cholangiocarcinoma, whose objective response rate in clinical trials are only 2.9 and 2%, respectively [12, 14]. These studies strongly suggest that precise clinical classification of patients is needed before treatment. Indeed, IDH mutations only drive the early stage of gliomagenesis [15], and are always accompanied with the secondary genetic abnormalities including tumor protein 53 (TP53) mutations, ATP-dependent helicase alpha-thalassemia syndrome protein (ATRX) loss, (RAS)-signaling pathway activation, chromosomal region 1p/19q co-deletion and so on [16, 17]. These alterations often become the leading causes of gliomagenesis in the late stage, and therefore should be considered as the foundations for the histological classification to improve the therapeutic response. Also, these secondary variations are the theoretical basis for novel therapeutics, including synthetic lethality and drug combination. Secondly, some nonnegligible drug-related adverse effects such as differentiation syndrome, leukocytosis and nausea also weaken the clinical value of these two drugs [11–13, 18]. Last, and most important of all, acquired clinical resistance has been reported in AML patients treated with enasidenib and ivosidenib due to the secondary mutations in the allosteric pocket of mIDH1/2 [19, 20]. Therefore, there is an urgent need for discovering novel therapeutic methods to overcome the problems above in IDH-mutated cancers [21, 22].

Drug combination is an available method to produce synergetic effect for treating disease with complicated pathogenesis and several druggable targets [23]. This synergism modulates these targets simultaneously, enhancing the susceptibility of pathogen to multi-drug therapy and overcoming the drug resistance originated from the single-target treatment [24]. In addition, the concept of synthetic lethality has been paid extensive attention due to its successfully application in oncotherapy, such as the poly (ADPribose) polymerase (PARP) inhibitor Olaparib, which is the most famous example of synthetic lethality between BRCA1/2 and PARP in the DNA repair pathway. It has been approved by FDA for the treatment of ovarian cancer and breast cancer carrying BRCA mutation [25]. It is desirable that more and more synthetic lethal interactions are being or waiting to be discovered by using genome-wide RNA interference (RNAi) screening or pharmacology methods [26, 27].

Besides the epigenetic reprogramming, R-2-HG has also been found to be involved in the abnormal regulation of other physiological processes, such as cellular metabolism, cellular apoptosis, redox homeostasis, cell cycle, DNA damage response, immune response and so on. These correlations are basic to the synthetic lethal interactions and synergetic therapies associated with mIDH. In this review, we first enumerate the targets exhibiting relevant synthetic lethal interactions with mIDH1/2. In detail, the inhibitors of these targets could suppress the growth or induced the redifferentiation of tumor cells expressing mIDH1/2. The mechanisms of these synthetic lethal interactions are also well elucidated. Then the compounds which displayed synergetic effect with mIDH1/2 inhibitors are also listed, especially the molecules combined with enasidenib or ivosidenib. We proposed that in the future these promising strategies would be applied in the clinic for the treatment of tumor harbor IDH mutations to combat the defects of existing mIDH1/2 inhibitors.

## Synthetic lethality in IDH-mutated tumors

**Fig. 3:**
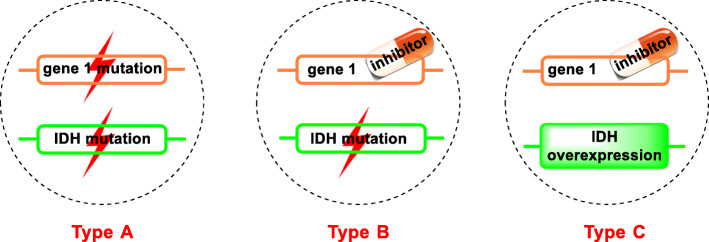
Three types of synthetic lethality associated with IDH. The orange and green frames represent two genes. The shape of lightning means gene mutation. Inhibitor here indicates the low expression of one gene

Synthetic lethality describes a phenomenon that the concurrent defects of two genes result in cell death, whereas a defect in either of them has little or no effect on cell viability [28]. Base on different principles, synthetic lethality of IDH can be classified into three types (Fig. 3) [26], the mutations in both genes (Type A), IDH mutation plus another gene’s inhibition (Type B), and IDH overexpression plus another gene’s inhibition (Type C).

In recent years, increasing studies have uncovered that IDH-mutated tumors are sensitive to the inhibition of several other targets. For example, the physiological process involved in DNA repair machinery was known to display synthetic lethal interaction with mIDH, making the tumor cells sensitive to radiation or chemotherapy [29]. It was reported that the glioma cells transduced with IDH1^R132H^ or IDH2^R172K^ exhibited increased cytotoxic actions to radiation [30]. Further, tumor cells expressing IDH1^R132H^ showed more susceptibility to chemotherapeutic agents such as temozolomide (TMZ) and cis-diamminedichloroplatinum (CDDP) in a time- and dose-dependent manner [31]. Collectively, the potential therapeutic efficacy will spur scientists to identify more synthetic lethal partner genes for IDH.

### IDH and glutaminase

Mitochondrial glutaminase (GLS) is often in charge of translating glutamine into glutamate and serving as a key role in cell metabolism, growth, and proliferation [32]. It has been widely accepted that cancer cells exhibited “glutamine addiction”, with the overexpression of GLS and rapid dying after glutamine depletion [33]. Given its broad role in energetic and biomass requirements for rapidly proliferating cells, GLS has become an attractive target for cancer therapeutics. Telaglenastat (CB-839), the first-in-class, selective, and orally active GLS inhibitor developed by Calithera Biosciences, is now undergoing clinical trials for the treatment of advanced solid tumors and hematological malignancies [34].

It was reported that the tumor cells expressing mutations in IDH1/2 often appear distinct metabolic characteristics, especially the strong dependency on glutamine as the main cellular source of *α*-KG. In the meantime, the generated *α*-KG would be reduced to R-2-HG with NADPH and Mg^2+^ as the cofactors [35]. Additionally, the metabolic stress produced in diffuse glioma cells expressing IDH1^R132H^ can be alleviated by lactate and glutamate [36]. Therefore, glutamine is crucial for the survival of IDH-mutated tumor cells, and interfering with the glutamine metabolism by GLS inhibitors would result in the deficiency of *α*-KG, and hence the decreased level of R-2-HG. This would be a novel therapeutic strategy for cancers carrying IDH mutations. It is worth noting that GLS inhibitor BPTES, a lead compound of CB-839, could suppress the growth of five primary AML cells expressing mIDH1/2, but lacking of effectiveness in wild-type cells [37]. Apart from AML, glioma cells with IDH1^R312H^ also showed this metabolic synthetic lethality, exhibiting the preferential sensibility to glutaminase inhibitor. Moreover, inhibiting glutaminase by siRNA or BPTES markedly slowed the growth of IDH1-mutated glioblastoma cells when compared to those expressing wild-type IDH1 [38]. All these studies open a way for novel approaches to target a specific subtype of AML or glioma metabolically with IDH mutations and a unique reprogramming of intermediary metabolism that culminates in glutamine dependency of cancer cells for survival.

### IDH and DOT1L

Disruptor of telomeric silencing 1-like (DOT1L) is a histone lysine methyltransferase (HMT) that specifically catalyzes the mono-, di-, and tri-methylation of the histone H3-lysine79 (H3K79) [39]. DOT1L-mediated H3K79 methylations play an important role in transcriptional elongation, cell cycle regulation, and DNA damage response [40]. The aberrant methylation of H3K79 by DOT1L would enhance the expression of leukemogenic genes homeobox A9 (HOXA9) and Meis Homeobox 1 (MEIS1), driving the pathogenic of mixed lineage leukemia (MLL) rearranged leukemia [41]. At this point, DOT1L has been found to be an important drug target for the therapy of acute leukemia with MLL gene translocations, and several kinds of DOT1L inhibitors were discovered in the past decade [42].

It was found that the global levels of H3K79 dimethylation (H3K79me2) were abnormally increased in IDH1/2-mutated tumor cells due to the overexpression of DOT1L [43]. Ley group from Washington University rendered DOT1L inhibitors to primary AML cells carrying IDH1/2 mutations to investigate whether these molecules could also be active in AML cases that lacking MLL rearrangements [44]. To its surprise, the selective DOT1L inhibitor EPZ004777 induced redifferentiation associated with apparent morphologic changes of these AML cells after 10 days’ incubation, which indicated the synthetic lethal-like effect resulted from mIDH and DOT1L. Nevertheless, this effect didn’t lead to the death of AML cells. It appears that treating IDH mutated tumor with DOT1L inhibitor may also be a feasible strategy for AML.

### IDH1 and Bcl-2/Bcl-xL

Since the initial discovery in the context of B-cell lymphoma (Bcl) in the 1980s, the Bcl-2 family has long been identified as the apoptosis-suppressing oncogene. Based on different structural and functional features, the Bcl-2 family is divided into three subfamilies, including anti-apoptotic proteins, pro-apoptotic (BH3-only) proteins, and pore-forming or ‘executioner’ (pro-apoptotic) proteins [45]. The anti-apoptotic subfamily contains several members such as Bcl-2, Bcl-xL, BCL-w, BCL-B, MCL-1, and Bfl1/A-1 [46]. Among them, Bcl-2 is the first identified member due to its important role in B-cell lymphoma [47]. Bcl-2, as well as Bcl-xL, were found overexpressed in various tumors [48]. As a druggable target, the first Bcl-2 inhibitor venetoclax was approved by FDA in 2016 for the treatment of chronic lymphocytic leukemia (CLL) or small lymphocytic leukemia (SLL) in adults [49].

In 2015, Chan and his co-workers identified Bcl-2 as a target that can form synthetic lethal interaction with IDH1^R132H^ using a large-scale RNAi screening [50]. The results showed that primary human AML cells with IDH1/2 mutation were more sensitive to venetoclax in both ex vivo and in vivo models than the wild-type cells. Also, the oncometabolite R-2-HG suppressed the activity of cytochrome c oxidase (COX) in the mitochondrial electron transport chain (ETC), subsequently lowered the mitochondrial threshold to trigger apoptosis upon Bcl-2 inhibition, which accounts for the enhanced sensitivity of AML cells to venetoclax. This synthetic lethal interaction constructs the rational basis for combining agents with venetoclax to target resistant cancer cells and maximize the clinical utility of this promising drug.

Similarly, researchers from Columbia University Medical Center have identified Bcl-xL as another target that held synthetic lethal interaction with IDH1^R132H^ [51]. They found U87MG and T98G glioblastoma cells with IDH1^R132H^ mutation displayed significantly higher sensitivity to the Bcl-xL inhibitor ABT263 than the wild-type counterparts. Moreover, the glioblastoma cells treated with ABT263 showed a markedly enhanced cleavage of caspases 9, 3, and PARP, which well explained the mechanism responsible for the enhanced anti-proliferative response toward the Bcl-xL inhibitor. These results support more therapy choices for IDH1-mutated gliomas.

### IDH and PARP

As the important DNA damage sensors and signal transducers, PARP enzymes are essential for the repair of single-strand DNA breaks (SSBs) and other types of DNA lesion via the base excision repair pathway [52]. The activated PARP triggers DNA damage response (DDR) that stall the cell cycle and mediate DNA repair, thus maintaining the integrity and stability of the genome in healthy cells. Besides that, BRCA1/2 plays a critical role in the cellular response to DNA double-strand breaks (DSBs) by the homologous recombination (HR) pathway [53]. Mutations in BRCA1/2 would lead to the impairment of the HR pathway and therefore unable to repair the DSBs [54]. Further, inhibition of PARP causes an increase of SSBs, and subsequently the rise of DSBs because of the replication fork collapse, showing synthetic lethal effect as well as inducing the cell death in BRCA1/2-mutated tumors [55].

Sulkowski et al. discovered a markedly reduced capacity to repair DSBs in several genetically diverse cell lines with IDH1 mutation [56]. It also suggests that mutations in IDH1/2 always induce HR defect that renders tumor cells exquisitely sensitive to PARP inhibition. The R-2-HG mediated direct inhibition of αKG dependent dioxygenases (especially KDM4A and KDM4B) are the main reasons for HR suppression. In total, this “BRCAness” phenotype was originated from synthetic lethal interaction between mIDH1/2 and the inhibition of PARP.

### IDH1 and mTORC1

The mammalian target of rapamycin (mTOR) is a large serine/theronine protein kinase which is activated by the PI3K/Akt signaling pathway [57]. It is involved in several physiological processes, such as regulating cellular metabolism, cellular growth, protein synthesis, proliferation and survival, whose overactivation is closely associated with the initial and progression of cancer [58]. Thus, mTOR has been regarded as an effective target for cancer therapy [59]. Actually, mTOR resides in two distinct multiprotein complexes: the rapamycin- sensitive complex (mTORC1) and the rapamycin-resistant complex (mTORC2). The mTORC1 is responsible for modulating the translation, ribosome and mitochondrial biogenesis, autophagy, lipid biosynthesis via integrating signals from cell surface receptors, energy status, stress, and availability of oxygen and amino acids [60]. As an inhibitor of mTORC1, rapamycin exhibits potential therapeutic effects on age-related diseases, such as cancer, neurodegenerative disease, and type 2 diabetes [61].

Hujber and co-workers found the IDH1-mutated tumor cells displayed remarkable sensitivity to mTORC1 inhibitor [62]. It was reported that rapamycin could reduce the production of lactate and R-2-HG in fibrosarcoma cells via inhibiting LDH-A and glutaminase, respectively. Additionally, rapamycin also elicited robust inhibitory effects in the proliferation and migration of IDH1-mutated HT-1080 cells, as well as altered their metabolic activity. These results strongly suggest that targeting mTOR activity could be an effective and novel strategy to eliminate tumor with IDH1 mutations.

### IDH and SRC

The Src is a group of non-receptor tyrosine kinase families associated with several cellular processes, such as proliferation, migration, differentiation and survival. This family consists of 11 members, among which Src is a representative one, which is ubiquitously expressed in mammals [63]. Src has been found directly involved in the transformation and oncogenesis along with its overexpression and high activation in a wide variety of human cancers [64]. Thus, it has been intensively investigated as a target for anti-tumor and several Src inhibitors have exerted fine therapeutic efficacy in the suppression of tumor growth and angiogenesis [65].

Dasatinib is the first Src/Abl dual inhibitor approved by the FDA for the treatment of chronic myeloid leukemia and Philadelphia chromosome-positive acute lymphoblastic leukemia [66]. Saha et al. revealed that the IDH-mutated intrahepatic cholangiocarcinoma (ICC) cells exhibited sensitivity to dasatinib [67]. The inhibition of Src signaling was defined as the key factor for this sensitivity. What’s more, dasatinib could also cause rapid and widespread cell death in patient-derived xenografts with IDH mutation. Thus, this novel and dramatic synthetic lethal effect conferred by mIDH and SRC will be a potential treatment protocol for relevant cancers.

### IDH1 and GDH

Glutamate dehydrogenase (GDH) is in charge of catalyzing the reversible inter-conversion of L-glutamate to *α*-KG using ammonia using NAD(P)^+^ as a coenzyme [68]. This metabolic enzyme can be found in all living organisms, while high levels of GDH activity are always detected in mammalian liver, kidney, brain and pancreas. Emerging evidence indicated that GDH was significantly overexpressed across many cancer subtypes [69].

The metabolic reprogramming in IDH-mutated cells always induced high metabolic stress [70]. In order to overcome this abnormal stress, the mutated cell often adopts the rescue mechanisms, such as upregulating glutaminase and glutamate processing [36]. At this point, the inhibition of glutamate processing with GDH inhibitor epigallocatechin-3-gallate (EGCG) would decrease the synthesis of NAD(P)H and *α*-KG in IDH-mutated cancers, resulting in increased oxidative stress and metabolic stress, and subsequently improved sensitivity to radiotherapy [71]. Studies have validated that EGCG could significantly decrease the proliferation rates of HCT-116 colorectal cells with IDH1^R132H^ knock-in under ionizing radiation. However, this synthetic lethal effect was diminished when treating with mIDH1 inhibitor AGI-5198. It therefore appears that the synthetic lethal activity derived from mIDH1 and GDH may be an effective and promising option for IDH-mutated cancers.

### IDH and DLL3

Delta-like ligand 3 (DLL3) is an inhibitory Notch ligand that is minimally expressed in normal tissues but always specially and highly expressed on the surface of small cell lung cancer (SCLC) cells and other high-grade neuroendocrine tumor cells [72, 73]. Researches have indicated that Notch could be inhibited by overexpressed DLL3, which would exert an important role in the onset and progression of cancer [74]. At this point, DLL3 has been defined as a potential therapeutic target in SCLC or neuroendocrine tumors [75]. Rovalpituzumad is a first-in-class antibody-drug conjugate directed against DLL3 for the treatment of SCLC [76]. Spino et al. reported that IDH mutated glioma cells showed significantly higher expression of DLL3 than wild-type cells. What’s more, the patient-derived IDH-mutated glioma displayed robustly sensitivity to rovalpituzumad in an antigen-dependent manner [77]. These considerations highlight that DLL3 inhibition may be a favorable therapeutic strategy for the cure of glioma with IDH mutation.

### IDH1 and Nrf2

Glutathione (GSH) is responsible for defending against oxidative stress in all mammalian cells. As the key determinant of redox signaling, GSH is vital in detoxification of xenobiotics and modulating cell proliferation, apoptosis, immune function, and fibrogenesis [78]. The abnormal levels of GSH are known to contribute to the initial and development of many pathological conditions, such as diabetes mellitus, pulmonary and liver fibrosis, alcoholic liver disease, drug-resistant tumor and so on. Glutamate cysteine ligase (GCL) and GSH synthetase (GS) are two important enzymes in GSH synthesis, whose activity is always regulated at multiple levels in a coordinated manner [79]. Among them, NF-E2 related factor 2 (Nrf2) is the key transcription factor that regulate the expression of these genes via the antioxidant response element (ARE) [80].

Xu group found reactive oxygen species (ROS) and the oxidative damage were remarkable accumulation in IDH1-mutated cells [81]. Consistently, the cells harboring IDH1 mutations exhibited enhanced transcriptional activity of Nrf2 along with the elevation of oxidative stress, which would raise the expression of GLC subsequently. Thus, blockading Nrf2 activation by genetic or pharmacologic methods could not only disrupt ROS homeostasis but also enhance oxidative DNA damage and decrease the proliferation of IDH1-mutated cells [82–84]. Triptolide, a potent Nrf2 inhibitor, has been shown to effectively suppress patient-derived IDH1-mutated glioma cells and exhibited selective cytotoxicity to preclinical glioma xenograft with IDH1 mutations [81]. In general, this synthetic lethality derived from IDH1 mutants and Nrf2 displayed the therapeutic potential in refractory malignancies.

### IDH1 and H3K27M

As the mark characteristic for facultative heterochromatin, H3K27 trimethylation (H3K27me3) is associated with transcriptionally repressed regions [85]. Polycomb repressive complex 2 (PRC2) is responsible for depositing, recognizing and propagating H3K27 methylation to maintain the silent state of the genes during development and cell differentiation [86]. Previous studies have identified the H3K27 mutation (H3K27M) in more than 80% diffuse intrinsic pontine gliomas (DIPGs) [87], which is fatal and lacks effective treatments in childhood brain tumors [88]. H3K27M showed a strong affinity for methyltransferase EZH2, the catalytic subunit of the PRC2, repressing H3K27me3 and thereby inhibiting PRC2’s enzymatic activity. Among histone H3, H3.3 is a major variant, and the K27M mutation in histone H3.3 (H3.3K27M) has been identified as a driver mutation for DIPGs [89]. The mutant cells showed decreased levels and altered distribution of H3K27 trimethylation (H3.3K27me3) via multiple mechanisms, including aberrant PRC2 interactions and hampered H3.3K27me3 spreading.

The cell harbor H3.3K27M mutation showed a distinct metabolic pattern, such as enhanced glycolysis, glutaminolysis, and TCA metabolism with high *α*-KG production [90]. Nevertheless, *α*-KG often displays contrary level of quantity with H3K27me3 in H3.3K27M cells [91]. The low level of *α*-KG production by inhibiting the key enzymes in glycolysis or glutaminolysis showed survival benefit in animal models. It was noteworthy that mIDH glioma cells consumed excessive *α*-KG and subsequently resulted in the increased level of H3K27me3. Moreover, Chung and co-workers found that H3.3K27M and mIDH1 adopt the opposite ways to hold a critical and conserved metabolic pathway and these two mutants displayed mutually exclusive and experimental synthetic lethality [91]. To inhibit IDH1 by shRNA or pharmacological methods in H3.3K27M cells could increase H3K27me3 levels and suppress cell proliferation duo to the altered chromatin accessibility at gene loci related to neuroglial differentiation. Ultimately, the therapeutic potential in preclinical DIPG animal models was presented when combined inhibition of *α*-KG-producing enzymes in glucose and glutamine metabolism pathways in DIPG animal models.

## Synergetic therapies in IDH-mutated tumors

### mIDH inhibitors and chemotherapeutic agents

Azacitidine (AZA) is a hypomethylating agent that prevents methylation by covalently binding DNA methyltransferases [92]. The mIDH1 inhibitor ivosidenib has the similar mechanism of action, which inhibits the hypermethylation of DNA and histone. Therefore, the combination of them has been considered to obtain more clinical benefits than monotherapy [93]. Indeed, preclinical work has shown that combination of ivosidenib and AZA could enhance mIDH1 inhibition-related redifferentiation or apoptosis in IDH1-transformed leukemic cell line [94]. On this foundation, an open-label, multicenter, phase Ib trial (NCT02677922) to evaluate safety and efficacy of combining ivosidenib with AZA in patients with newly diagnosed IDH1-mutated AML ineligible for intensive induction chemotherapy was performed [95]. This combination was proved well tolerated, with an expected safety profile consistent to monotherapy. Preliminary efficacy data were promising in difficult-to-treat patients, with the deep and durable remissions and the clearance of IDH1 mutation in the majority of responders.

Apart from this combination above, ivosidenib is also discovered in the clinical trials of combinatorial therapy with PD-1 inhibitor nivolumab (NCT04056910), the dual-drug liposomal encapsulation of cytarabine and daunorubic in CPX-351 (NCT04493164), decitabine/cedazuridine and BCL-2 inhibitor venetoclax (NCT04774393, NCT03471260), the chemotherapeutics such as cytarabine or fludarabine (NCT04250051), and cisplatin (NCT04088188). Also, enasidenib is investigated to combine with AZA (NCT03683433, NCT03383575), JAK inhibitor ruxolitinib (NCT04281498), venetoclax (NCT04092179), CPX-351 (NCT03825796), decitabine/cedazuridine and venetoclax (NCT04774393). Unfortunately, these clinical trials are under recruiting or not yet recruiting thereby no available data are disclosed (Table 1). However, these combined pharmacotherapies would represent an important tendency for the treatment AML or other tumors.

**Table 1:** Overview of selected clinical trials of combined pharmacotherapy between mIDH1/2 inhibitors and other molecules

Drug combination		Phase	Number Enrolled	Inclusion criteria	Trail registration	Status
**Ivosidenib**	AZA	Ib/II	131	Newly diagnosed AML	NCT02677922	Active, not recruiting
	venetoclax	II	48	AML	NCT03471260	Recruiting
	FLAG Chemotherapy	I	25	R/R AML	NCT04250051	Not yet recruiting
	CPX-351	II	30	AML/MDS	NCT04493164	Recruiting
	gemcitabine + cisplatin	I	40	Cholangiocarcinoma	NCT04088188	Recruiting
	Decitabine/ ASTX727 + venetoclax	Ib/II	84	R/R AML	NCT04774393	Not yet recruiting
**Enasidenib**	AZA	II	50	R/R AML	NCT03683433	Recruiting
	AZA	II	105	MDS	NCT03383575	Recruiting
	Ruxolitinib	II	32	Myeloproliferative neoplasm	NCT04281498	Not yet recruiting
	CPX-351	II	18	Relapsed AML	NCT03825796	Recruiting
	venetoclax	Ib/II	48	AML	NCT04092179	Recruiting
	Decitabine/ ASTX727 + venetoclax	Ib/II	84	R/R AML	NCT04774393	Not yet recruiting

### mIDH and radiotherapy/chemotherapy strategies

Like normal cells, tumor cells constantly suffer DNA damages every day and insufficient repairs will drive them towards apoptosis. Therefore, a complete DNA repair system is needed to prevent the cytotoxic or mutagenic effects derived from various types of DNA lesions. As the major enzyme for repairing the methylated lesions on DNA and RNA, the function of *α*-KG -dependent dioxygenase AlkB superfamily is known to remove *N*-methyl groups preferentially from 1-methyladenine or 3-methylcytosine in single-stranded DNA [96]. Human cells consist nine distinct ALKB homologs (ALKBH), namely ALKBH1–8 and fat mass and obesity-associated protein (FTO) respectively [97]. Notably, ALKBH2 and ALKBH3 have been shown to catalyze nucleic acid *N*-demethylation, repairing DNA alkylation lesion preferentially [98].

As a Fe^2+^ and *α*-KG-dependent dioxygenase, the activity of AlkB was reported to be inhibited by R-2-HG [99]. It therefore appears that tumor cells with IDH1 mutation display more sensitive to alkylating agents than wild-type cells due to the reduced repair kinetics and increased DNA damages. On the other hand, R-2-HG could elicit the downregulation of DNA damage sensor ataxia telangiectasia mutated (ATM) and subsequently increase the impairment of DNA repair, finally enhance the sensitivity of tumor cells to alkylating agents [100]. Typically, AML cells or glioma cells harbor IDH mutations showed increased sensitivity to radiotherapy and chemotherapy [101, 102]. It is interesting that the deletion of mIDH1 allele or overexpression of ALKBH2 or AKLBH3 could reverse this synergetic effect. These results strongly suggest that the mIDH1/2 derived impairment of DNA repair might contribute to tumorigenesis and classical alkylating agents may offer a way forward the “targeted” therapy for patients with IDH-mutated cancers.

### mIDH1 inhibitor and TET inducer

As a hallmark of cancer, the disruption of epigenetic landscapes such as the changes of DNA methylation patterns, are closely associated with the pathogenesis of tumor [103, 104]. TET is known to catalyze the demethylation of 5-methylcytosine (5mC) [105]. It contains three subunits, TET1, TET2, and TET3, that catalyze the successive oxidation of 5-methylcytosine to 5-hydroxymethylcytosine (5hmC), 5-formylcytosine (5fC), and 5-carboxylcytosine (5caC) [106]. Several studies have recognized TET enzymes as the important tumor suppressors and the mutants in all three TET genes were uncovered [107]. They showed reduced expression and impaired activity in a wide range of different cancer types [108].

TET is an *α*-KG dependent dioxygenase and its activity could be inhibited by mIDH1/2 derived R-2-HG, which leads to epigenetic alteration [109]. Gerecke et al. found that Vitamin C (VC), the inducer of TET enzymes, could obviously reduce R-2-HG to the levels comparable to those in wild-type HCT116 cell line when combinatorial treating with mIDH1 inhibitor ML309 [110]. Additionally, this reduction of R-2-HG was accompanied by an enhanced global DNA hydroxymethylation and an increased gene expression of certain tumor suppressors. Most important of all, IDH1-mutated tumor showed an increased percentage of apoptotic cells after combining use ML309 and VC. It is also critical to note that high doses of VC can exert its anticancer effect via multiple mechanism of actions, including modulating infiltration of the tumor microenvironment by cells in immune system to delay cancer growth [111], accumulating ROS and inhibiting glyceraldehyde 3-phosphate dehydrogenase (GAPDH) to kill the colorectal cancer cells harboring KRAS or BRAF mutations [112], targeting redox homeostasis in pheochromocytomas and paragangliomas (PCPG) cells with low levels of succinate dehydrogenase B subunit (SDHB) to suppress metastatic lesion and prolong overall survival [113], and et al. Therefore, this synergetic therapy needs to be further investigated. Collectively, these results strongly illustrate that combinatorial therapy is of great interest to rescue TET activity and treat IDH1/2-mutated cancers.

### mIDH inhibitors and immune checkpoint inhibitors

Indeed, R-2-HG is not only the epigenetic modifier involved in neoplasia, but also an immunosuppressive metabolite. It has been reported that R-2-HG always accumulate in tumor environment and can be taken up by CD8^+^ T cells, hence impairing their capacity of anticancer immune responses [114]. Additionally, R-2-HG was also explored to inhibit the tumor-infiltrating lymphocytes and natural killer cells, causing enhanced immune suppression in tumor microenvironments [115, 116]. On the basis of these considerations, the inhibitors of mIDH actually play dual antitumor effect by causing the epigenetic reprogramming of malignant cells and relieving R-2-HG-mediated immunosuppression. From this it appears that combining cancer metabolism therapy with immunotherapy will be a prospective strategy for overcoming cancer [117], and in particularly, mIDH inhibitors should be paired with the immune checkpoint inhibitors to improve the clinical response of patients with IDH-mutated tumors [118].

It is worth noting that IDH1R132H inhibitor BAY1436032 collocated with PD-1 inhibitor would result in an increase of overall survival in C57BL/6 J mice bearing IDH1-mutated glioma [119]. To our knowledge, the phase II study involved in ivosidenib in combination with immune checkpoint inhibitor nivolumab for patients with IDH1-mutated R/R AML and high risk myelodysplastic syndrome (MDS) [120], advanced solid tumors or enhancing gliomas (NCT04056910) is carrying out. Unfortunately, there is no available data uncovered at present.

## Conclusions

As the druggable targets, mIDH1/2 have been paid much attentions due to their therapeutic potential as well as the swiftly successful in drug discovery. Nevertheless, the insuperable weaknesses of two approved inhibitors render researchers to look for alternative methods. With the successful application of PARP inhibitors, several screening programs using siRNA or pharmacological methods have been conducted to identify the targets that can generate synthetic lethal interaction with mIDH1/2 and the molecules can supply synergetic therapy with mIDH1/2 inhibitors. Indeed, these novel therapeutic strategies have exhibited significant effectiveness with the enhanced sensitivity to diverse tumor cells, especially AML and glioma with IDH mutations.

Despite strenuous efforts have been paid, there are still several challenges for transforming these synthetic lethal interactions and synergetic therapies into clinical benefits. The magnitude of a given synthetic lethal effect is the one of the major challenges. Some of the synthetic lethal interactions showed statistically significant and even biologically meaningful, but are unable to translate into clinic due to their small efficacy in the trails. Consider olaparib as a reference, whose synthetic lethal effect with mutant BRCA1/2 is about 100-fold difference in sensitivity when compared to those in wild-type cells. The second challenge is the heterogeneity of cancer [121], with many synthetic lethal targets only existed in a small number of cancer subtypes. In addition, this heterogeneity also makes a portion of cells response to synthetic lethal effect, while it is ineffective to other portion of cells. Actually, the biological targets of R-2-HG are more than 60 members in cells [122], which are involved in profoundly diverse molecular pathways. Thus, it remains not clear for the real oncogenic mechanisms of glioma and it will be a great challenge for applying the synthetic lethal effect to glioma. Other challenges for synthetic lethal effect include how to obtain cell lines stably expressing mutant IDH and avoid the false positive screening by using the novel techniques. Last, favorable blood-brain barrier (BBB) penetration of mutant IDH inhibitors and other drugs is the foundation of synthetic lethal interaction and synergetic therapies to induce anti-tumor effects in CNS tumors, especially for gliomas. In summary, we trust that induction of these therapeutic strategies into IDH-mutated neoplasms is feasible and potentially highly efficacious in future clinical treatment.

## Data Availability

Not applicable.

## Abbreviations

mIDH1/2: Mutant isocitrate dehydrogenase 1/2
α-KG: α-ketoglutarate
R-2-HG: R-2-hydroxyglutarate
TCA: Tricarboxylic acid cycle
AML: Acute myeloid leukemia
PARP: Poly ADPribose polymerase
RNAi: RNA interference
TMZ: Temozolomide
CDDP: Cis-diamminedichloroplatinum
GLS: Glutaminase
DOT1L: Disruptor of telomeric silencing 1-like
HMT: Methyltransferase
H3K79: Histone H3-lysine79
MLL: Mixed lineage leukemia
CLL: Chronic lymphocytic leukemia
SLL: Small lymphocytic leukemia
COX: Cytochrome c oxidase
ETC: Electron transport chain
SSBs: Single-strand DNA breaks
DDR: DNA damage response
DSBs: Double strand breaks
HR: Homologous recombination
mTOR: mammalian target of rapamycin
GDH: Glutamate dehydrogenase
5mC: 5-methylcytosine
PHD: Prolyl hydroxylase
EGCG: Epigallocatechin-3-gallate
DLL3: Delta-like ligand 3
SCLC: Small cell lung cancer
GSH: Glutathione
GCL: Glutamate cysteine ligase
GS: GSH synthetase
Nrf2: NF-E2 related factor 2
ARE: Antioxidant response element
ROS: Reactive oxygen species
H3K27me3: H3K27 trimethylation
PRC2: Polycomb repressive complex 2
DIPGs: Diffuse intrinsic pontine gliomas
AZA: Azacitidine
PI: Proteasome inhibitors
MM: Multiple myeloma
MCL: Mantle cell lymphoma
R/R AML: Relapsed/refractory acute myeloid leukemia
TP53: Tumor protein 53
ATRX: Alpha-thalassaemia syndrome protein
HOXA9: Homeobox A9
MEIS1: Meis Homeobox 1
Bcl: B-cell lymphoma
ICC: Intrahepatic cholangiocarcinoma
ATM: Ataxia telangiectasia mutated
MDS: Myelodysplastic syndrome
